# Dynamic Polarization Behaviors of Equimolar CoCrFeNi High-Entropy Alloy Compared with 304 Stainless Steel in 0.5 M H_2_SO_4_ Aerated Aqueous Solution

**DOI:** 10.3390/ma15196976

**Published:** 2022-10-08

**Authors:** Chao-Chun Yen, Ting-Lun Tsai, Bo-Wei Wu, Yu-Chieh Lo, Ming-Hung Tsai, Shiow-Kang Yen

**Affiliations:** 1 Department of Materials Science and Engineering, National Chung Hsing University, Taichung 40227, Taiwan; 2 Department of Materials Science and Engineering, National Yang Ming Chiao Tung University, Hsinchu 30010, Taiwan

**Keywords:** high-entropy alloy, first-principles calculations, polarization, ICP-MS, passive film

## Abstract

Three corrosion potentials and three corrosion current densities are clearly identified before the passivation for both dynamic polarization curves of equimolar CoCrFeNi high-entropy alloy (HEA) and 304 stainless steel (304SS) in 0.5 M H_2_SO_4_ aerated aqueous solution, by decomposing anodic and cathodic polarization curves. The passivated current density of the former is greater than the latter, compliant with not only the constant of solubility product (k_sp_) and redox equilibrium potential (*E_eq_*) of each metal hydroxide but also the sequence of bond energy (*E_b_*) for monolayer hydroxide on their facets derived from the first principle founded on density function theory. However, the total amount of ion releasing from HEA is less than 304SS, since the hydroxide/oxide film formed in the air of the latter containing greater amounts of Fe(Ⅱ) and Mn(Ⅱ) is less stable around corrosion potentials while they are further oxidized into more stable Fe(Ⅲ) and Mn(ⅢorⅣ) with much lower k_sp_, leading to the much less increasing ratios of ion releases from 0.25 to 0.6 V.

## 1. Introduction

High-entropy alloys (HEAs), unlike traditional single-element base ideas, were originally proposed as alloys composed of several major elements in equimolar or near-equal molar proportions [1,2]. By using this new concept of alloy design, the formation of intermetallic compounds can be curbed to urge the solid-solution phase(s) in the form of simple crystal structures including face-centered cubic (FCC), body-centered cubic (BCC), or hexagonal closed-pack (HCP) [3,4,5]. The concentrated solid-solution structures of HEAs often exhibit remarkable properties, including exceptional strength [6,7], high fatigue resistance [8], high thermal stability [9], superior electrical resistivity [10], and outstanding wear resistance [11]. However, most of the metal elements composing HEAs are thermodynamically unstable in the natural environment on the earth. Therefore, it is an important issue to design the HEAs for forming protective films to hinder or retard the environmental surrounding attacks.

It is well known that the nature of the passivation film consisting of a Cr-enriched oxide and/or hydroxide film determines the corrosion resistance of the stainless steel. King and Uhlig reported the polarization data by the adsorbed oxygen film composing the passivation film [12]. Aronowitz and Hackerman also consider it to be an absorbed single layer of oxygen [13]. The thickness of Fe-Cr alloy passivation film formed in the acidic solution ranging from 1.2~2.5 nm was analyzed by series techniques [14,15,16,17,18,19,20,21], including that Cr^3+^ oxide or hydroxide with a relatively small amount of Fe^2+^ is the major component on the film formed at a lower potential. Recently, E. Hamada reported that the thickness of passivation film formed on commercial 304 stainless steel after being kept in the atmosphere for a decade is from 3 to 5 nm [22]. Additionally, it has been shown that the adsorption of hydroxide ions on pure metals (copper, nickel, and chromium) and chromium-containing alloys (stainless steel) precedes the growth of three-dimensional oxides and produces a two-dimensional adsorbed hydroxyl covering layer acting as the structural precursor for the growth of three-dimensional passivation film [23].

Like stainless steel, most HEAs contain passivating elements that help form passivation layers, such as Al, Cr, Mo, and the like. The corrosion behavior of CoCrFeNi-based HEA has been much revealed [24,25,26,27,28,29]. Adding different elements in equimolar CoCrFeNi-X (X = Al, Cu, Sn) causes different corrosion resistance in a chlorine environment due to the various qualities of extensive metal oxides and galvanic corrosion between phases [24]. In 0.1 M H_2_SO_4_, the corrosion behavior of the equimolar CoCrFeMnNi HEA was compared to 304 stainless steel (304SS) because both single FCC phase metals have similar corrosion-resistance elements. The corrosion resistance of the HEA is worse than 304SS since the passivation film of HEA was formed by various elements and meanwhile the insufficient content of Cr [29]. In the latest three years, the corrosion mechanism of tri-phase equimolar AlCoCrFeNi high-entropy alloy has been reasonably explained by combining metal hydroxides reduction-oxidation potential derived by the Nernstian equation, the solubility product constant (k_sp_), and the compositional difference in three phases [28]. It has been reported that decreasing Ni content with increasing Fe or Co content in a single FCC phase Co_a_CrFe_b_Ni_80-a-b_ system could reduce passivation current density and lead to improving corrosion resistance [30], while the inverse effect of Ni was found on the corrosion resistance of TiZrHfBeCu (Ni) high-entropy bulk metallic glass in 3.5 wt.% NaCl [31]. Furthermore, tuning minor additions of Ti and Al to form (CoCrFeNi)_94_Ti_1.5_Al_4.5_ HEA has enhanced the corrosion resistance in 0.5M H_2_SO_4_ for the coating on Q235 steel substrate by plasma cladding [32]. Obviously, it is an important issue for upgrading the resistant performance of HEA against the corrosive environment. However, the corrosion behavior of equimolar CoCrFeNi HEA is rarely obtained, especially for the role of each element at various potentials during the polarization and passivation.

In this study, the potentiodynamic curves of 304SS and CoCrFeNi HEA in 0.5 M H_2_SO_4_ aerated aqueous solutions are quite similar. The alloy equilibrium potential (*E_eg_*) is calculated by the Nernst equation and Gibbs energy of individual metal, and the potentiodynamic curves are decomposed into anodic curves and cathodic curves to understand the reaction at each potential precisely. According to density function theory (DFT), the first-principles calculations have been used to design a new organic inhibitor for corrosion [33] or to investigate the adsorption of oxygen on metal surfaces [34,35]. In addition, it was also applied to assess the stability (the dissipated energy) of monolayer hydroxide on the tri-phase HEA [28]. Consequently, such a calculation conducted for the CoCrFeNi HEA and 304SS may also play a crucial role in the corrosion performance.

## 2. Materials and Methods

### 2.1. Sample Preparation

The CoCrFeNi HEA was manufactured by using Co, Cr, Fe, and Ni metal grains with high purity (99.99 wt%) according to molar ratios as primary materials and then placed in the inner side of a water-chilled Cu crucible and melted by arc in Ar atmosphere. The melting solidification process was repeated for three cycles to ensure the liquid homogenized completely, and then cooled to ambient temperature for deriving the as-cast ingot. The chemical compositions of CoCrFeNi HEA and commercial 304SS traded from the market characterized by energy dispersive spectroscopy (EDS) analysis are listed in Table 1, where elements such as C 0.06%, P 0.045% and S 0.015% less than 1% are neglected.

Test specimens were made by the wire cutting during electrical discharge then spot-welded with copper lines on the back side for the electrical conduction and finally cold-mounted in epoxy. The front side with exposed surface area 0.25 cm^2^ was wet ground by SiC papers ranging from grit number 100 to 2000, polished with alumina powder sized from 1 to 0.3 μm and cleaned thoroughly in a deionized water bath with an ultrasonic generator before each kind of electrochemical measurement.

### 2.2. Electrochemical Measurements

The electrochemical tests were carried out in a three-electrode cell. The working electrode was the specimen with surface area 0.25 cm^2^ immersed in 0.5 M H_2_SO_4_ aerated aqueous solution (AAS) at ambient temperature, using a commercial Ag/AgCl electrode in saturated KCl solution as the reference electrode (Ref) and a Pt plate was the auxiliary electrode. The potentiodynamic polarization measurement was conducted at scan rate 0.167 mV/s from initial potential −1.2 V to final potential 1.2 V versus the open current potential (OCP) (vs. *E_ocp_*) by Multi Potentiostat VSP and EC-lab software 11.35 (Bio-logic, Seyssinet-Pariset, France). Furthermore, the samples were kept at a constant potential of 0.6 V (vs. Ref) for 2 h to record the variation of current density for both metals.

### 2.3. Component Analyses of the Passivation Films

To evaluate the constituents and their related valences of the passivation films on the 304SS and equimolar CoCrFeNi HEA, which were formed in the air, at the first corrosion potential (*E_corr_*_1_) and 0.6 V (vs. Ref) in 0.5 M H_2_SO_4_ AAS, the specimens were washed with deionized water and dried before being transferred to the X-ray photoelectron spectroscopy (XPS) analyzer chamber, by using a ULVAC-PHI (Chigasaki, Japan), PHI 5000 VersaProbe/Scanning Electron Spectroscopy for Chemical Analysis (ECSA) Microprobe in conjunction with monochromatic Al Kα X-ray radiation with energy 1487 eV. The peaks of XPS data analyzed by XPSPEAK41 were also fitted by the mixed Gaussian–Lorentzian functions after Shirley background subtraction. The binding energy of peaks positions was consulted from NIST XPS Database.

### 2.4. Inductively Coupled Plasma Mass Spectrometry (ICP-MS)

To observe the dissolving elements on the CoCrFeNi HEA and 304SS in 0.5 M H_2_SO_4_ AAS, during polarization measurements and constant potential tests, 15 mL of the solution was taken from the corrosion cell for metal ion quantitative analyses by inductive coupled plasma mass spectrometry (ICP-MS) when the applied potential arrived at *E_corr_*_1_, 0.25 V and 0.6 V (vs. Ref), respectively.

### 2.5. First-Principles Calculations

Similar to the previous report [28], the electrochemical reactions on the surfaces of alloys in AAS are the following:

Anodic: Alloy → Alloy^n+^ + **n**e^−^ (1)

(2) $$\mathrm{Cathodic}:\;\frac{n}{4}O_{2}+\frac{n}{2}H_{2}O+ne^{-}\to n{\mathrm{OH}}^{-}$$

(3) $$\mathrm{The}\;\mathrm{net}:\;\mathrm{Alloy}+\frac{n}{4}O_{2}+\frac{n}{2}H_{2}O\to n\mathrm{OH}/\mathrm{Alloy}$$

The bond energy *E_b_* is defined as below

(4) $$E_{b}^{B}=-\frac{1}{n}\left(E_{OH/Alloy\;}-E_{Alloy\;}-\frac{n}{2}E_{H_{2}O}-\frac{n}{4}E_{O_{2}}\right)$$

(5) $$E_{b}^{A}=-\frac{1}{A}\left(E_{OH/Alloy\;}-E_{Alloy\;}-\frac{n}{2}E_{H_{2}O}-\frac{n}{4}E_{O_{2}}\right)$$

where $E_{b}^{B}$ is the bond energy per OH bond and $E_{b}^{A}$ is the bond one per surface area, ***n*** is the total number of electrons escaping from the alloy to form OH^−^ ions on its surface and n^+^ the positive charges left on alloy. Additionally, $E_{OH/\mathrm{Alloy}\;}$ and $E_{Alloy\;}$ are the total energies of the corresponding clean alloy slab and additionally containing the ad-layer of n hydroxyl ions on the slab and $E_{H_{2}O}$ and $E_{O_{2}}$ the energies per H_2_O, and O_2_ molecule, respectively, positive *E_b_* indicating the exothermic reaction at T = 0 K.

The calculated $E_{Alloy}$ and $E_{OH/Alloy}$ of CoCrFeNi and 304SS alloy including facets (100), (110), and (111) were conducted by the first-principal method based on DFT. As used in the previous report [29], the technical of special quasi-random structure (SQS) [36,37], the Vienna ab initio simulation program (VASP) [38,39], the projector augmented wave (PAW) method [40,41], and the exchange-correlation function as the generalized gradient approximation (GGA) parameterized by Perdew, Burke, and Ernzerhof (PBE) [42] were also included.

The electrons on semi-core p state were regarded as valence ones. The bulk calculation of alloy plane-wave energy cutoff was carried out by 600 eV with Monkhorst–Pack [43] 4 × 4 × 1 k-point mesh in the slab. The tolerance energy and atomic force were 1.0 × 10^−5^ and 1.0 × 10^−4^ eV, respectively, for the fully relaxed position, using SQS supercell composed of 144 atoms for both alloys to optimize the randomness of arrangement. The asymmetric slab composed of several atomic layers including half fixed bottom layers and half fully relaxed outer layers was employed to model all surfaces. For deriving $E_{OH/Alloy}$, the monolayer hydroxyl ions were set on each facet with the same stacking way as the original alloy layer and a vacuum distance of 12~15 Å from the top surface was assumed to avoid the coupling of consecutive slabs.

## 3. Result and Discussion

### 3.1. Electrochemical Corrosion Behaviors Analysis

**Figure 1:**
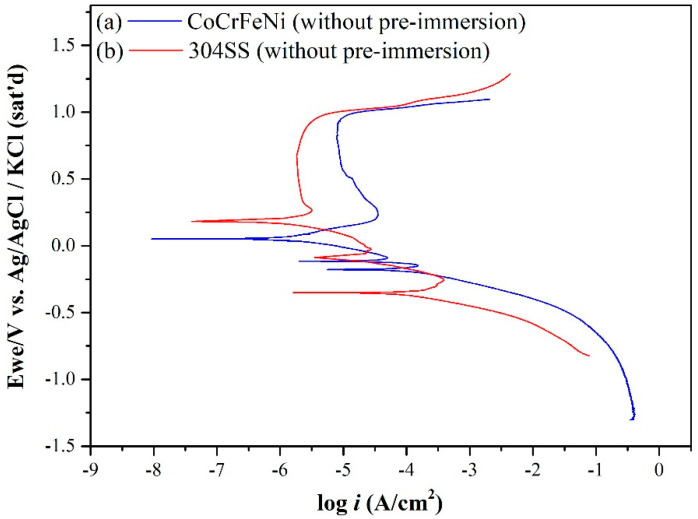
Potentiodynamic polarization curves of (a) CoCrFeNi HEA and (b) 304SS without pre-immersion in 0.5 M H_2_SO_4_ (pH = 0.47) AAS.

Figure 1 shows the dynamic polarization curves of equimolar CoCrFeNi HEA and 304SS specimens in 0.5 M H_2_SO_4_ AAS without pre-immersion, respectively. Table 2 presents the corrosion characteristics derived from Figure 1, including three corrosion potentials (*E_corr_*_1_, *E_corr_*_2_, and *E_corr_*_3_), three corrosion current densities (*i_corr_*_1_, *i_corr_*_2_, and *i_corr_*_3_), the passivation current density (*i_pass_*), the first critical current density (*i_crit_*_1_), the second critical current density (*i_crit_*_2_), and the trans-passive potential (*E_trans_*). Around *E_corr_*_1_, the metal elements may be initially oxidized into hydroxides on the surface as follows:

Ni(OH)_2_ + 2e^−^ = Ni + 2OH^−^         *E_eq_* = −0.115 V (vs., Ref) … (6)

Co(OH)_2_ + 2e^−^ = Co+ 2OH^−^         *E_eq_* = −0.125 V (vs., Ref) … (7)

Fe(OH)_2_ + 2e^−^ = Fe + 2OH^−^         *E_eq_* = −0.271 V (vs., Ref) … (8)

Cr(OH)_3_ + 3e^−^ = Cr + 3OH^−^         *E_eq_* = −0.574 V (vs., Ref) … (9)

Mn(OH)_2_ + 2e^−^ = Mn + 2OH^−^         *E_eq_* = −0.947 V (vs., Ref) … (10)

where *E_eq_* is the calculated reduction-oxidation equilibrium potential of metal and metal hydroxide derived from Nernst equation at pH = 0.47 for 0.5 M H_2_SO_4_ AAS, also listed in Table 3. The *E_corr_*_1_ of 304SS (−354 mV) is lower than that of HEA (−179 mV), consistent with *E_eq_*_304*SS*_ (−359 mV) lower than *E_eq CoCrFeNi_* (−305 mV). The less k_sp_ means the more stable hydroxide compound in aqueous solutions. The Cr content should play an important role in the first critical current density (*i_crit_*_1_), because the k_sp_ of Cr(OH)_3_ is much less than the other metal hydroxides including Mn(OH)_2_, Ni(OH)_2_, Co(OH)_2_, and Fe(OH)_2_, as listed in Table 4 [44]. Both the *i_crti1_* and *i_corr_*_1_ of CoCrFeNi HEA smaller than 304SS are straightforwardly ascribed to the higher Cr content of the former.

Since the k_sp_ values of iron (II), Mn(II) and cobalt(II) hydroxides are dramatically reduced from 2 × 10^−13^ and 3 × 10^−16^ to 6 × 10^−38^ and 1 × 10^−43^ for iron(III) and cobalt(III) ones, listed in Table 4 [40]. Fe, Co, Mn and Ni, beside Cr, may also play a role in the passivation zone by the following electrochemical reactions:

Fe(OH)_3_ + e^−^ = Fe(OH)_2_ + OH^−^         *E_eq_* = 0.048 V (vs., Ref) … (11)

Mn_3_O_4_ + 2H^+^+ H_2_O + 2e^−^ = 3Mn(OH)_2_         *E_eq_* = 0.237 V (vs., Ref) … (12)

Co(OH)_3_ + e^−^ = Co(OH)_2_ + OH^−^         *E_eq_* = 0.464 V (vs., Ref) … (13)

Co(OH)_3_ + e^−^ = Co(OH)_2_ + OH^−^         *E_eq_* = 0.773 V (vs., Ref) … (14)

2MnO_2_ + 2H^+^ + 2e^−^ = Mn_2_O_3_ + H_2_O         *E_eq_* = 0.789 V (vs., Ref) … (15)

Ni_2_O_3_ + 2H^+^ + H_2_O + 2e^−^ = 2Ni(OH)_2_         *E_eq_* = 0.807 V (vs., Ref) … (16)

However, Ni(OH)_2_ could not be further oxidized in reaction (16) at the potential below 0.8 V, keeping its k_sp_ value of 2 × 10^−16^, much greater than those of iron (III) and cobalt (III) hydroxides. The more Fe and Co content in the passivation film, the more anti-corrosion ability the alloy has, while the former in reaction (11) occurs at a lower potential than the latter in reaction (14). This means that the more Fe content in the alloy, the lower the potential for initiating and stabilizing passivation, and the less Ni content in the alloy, the more stable the passivation film, also resulting in less *i_pass_* and *i_crit_*_2_ for 304SS than that for CoCrFeNi HEA in 0.5 M H_2_SO_4_ AAS, as shown in Figure 1 and listed in Table 2.

The *E_corr_*_1_ (−0.354 V) of 304SS is very close to *E_eq_*_304*SS*_ (−0.359 V) calculated in Table 3, indicating some more active reactions should be considered such as metal–metal ion ones below or around *E_corr_*_1_, as listed the lower parts in Table 3 with the calculated reduction-oxidation equilibrium potential ($E_{eq}^{’}$) for the metal–metal ion reactions. It should be noticed that the $E_{eq}^{’}$ for alloys cannot be directly added or subtracted [45]; they should be first transformed to Gibbs energy by ∆G = −nF$E_{eq}^{’}$. Assuming all the metal ionic activities are 10^−6^ M, the potential-pH diagrams [46] show Co as Co^2+^, Cr as Cr^3+^, Fe as Fe^2+^, Ni as Ni^2+^, Mn as Mn^2+^ and Si as H_2_SiO_4_ at the corrosion potentials of CoCrFeNi HEA and 304SS with pH = 0.47 in 0.5 M H_2_SO_4_ AAS. According to $E_{eq}^{’}$ of the individual alloying elements as listed in Table 3, the calculated equilibrium potentials of CoCrFeNi HEA ($E_{eq\;CoCrFeNi}^{’}$) and 304SS ($E_{eq\;304SS}^{’}$) in 0.5 M H_2_SO_4_ AAS are derived as follows:

(17) $$E_{eq,\;CoCrFeNi}^{’}=[(-0.457\times 2\times 24.36/100)+(-0.864\times 3\times 26.85/100)+(-0.627\times 2\times 25.18/100)+(-0.430\times 2\;\times 23.61/100)]÷226.85/100=-0.634\;\mathrm{VSHE}=-0.831\;V\;(\mathrm{vs.},\;\mathrm{Ref})\ldots$$

(18) $$E_{eq\;304SS}^{’}=[(-0.627\times 2\times 68.65/100)+(-0.864\times 3\times 20.65/100)+(-0.430\times 2\times 7.5/100)+(-1.356\times 2\times \;2.12/100)+(-1.096\times 4\times 1.08/100)]÷222.81/100=-0.703\;\mathrm{VSHE}=-0.900\;V\;(\mathrm{vs.},\;\mathrm{Ref})\ldots$$

**Figure 2:**
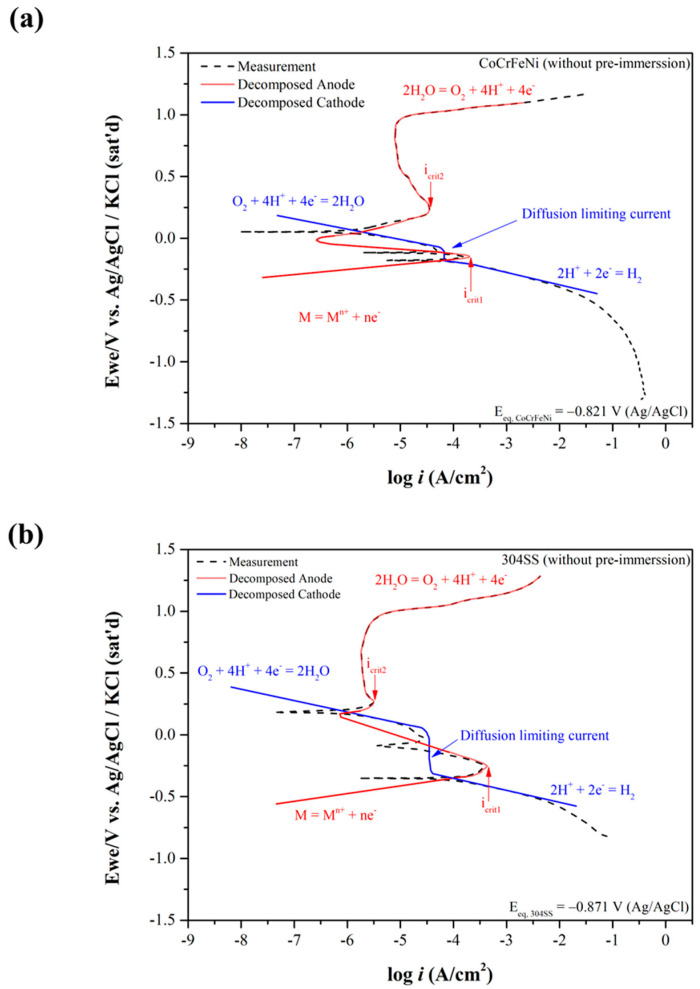
The anodic curves and cathodic curves decomposed from Figure 1 for (**a**) CoCrFeNi HEA and (**b**) 304SS.

**Figure 3:**
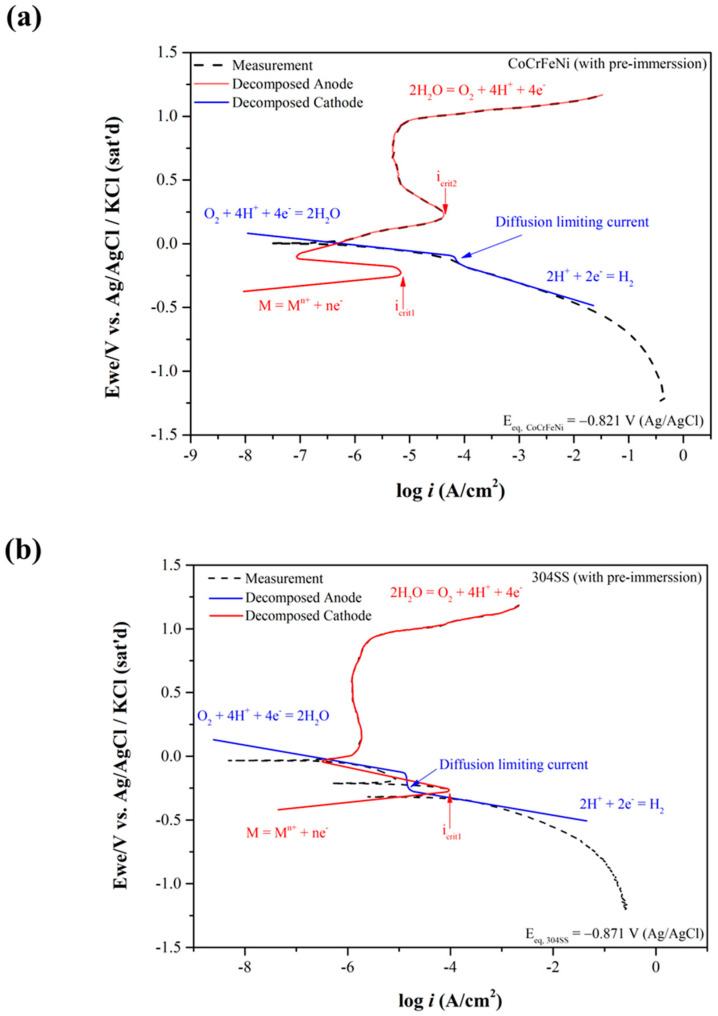
Dynamic polarization curves of (**a**) CoCrFeNi HEA and (**b**) 304SS with pre-immersion for 30 min, also decomposed into the anodic and cathodic ones.

**Figure 4:**
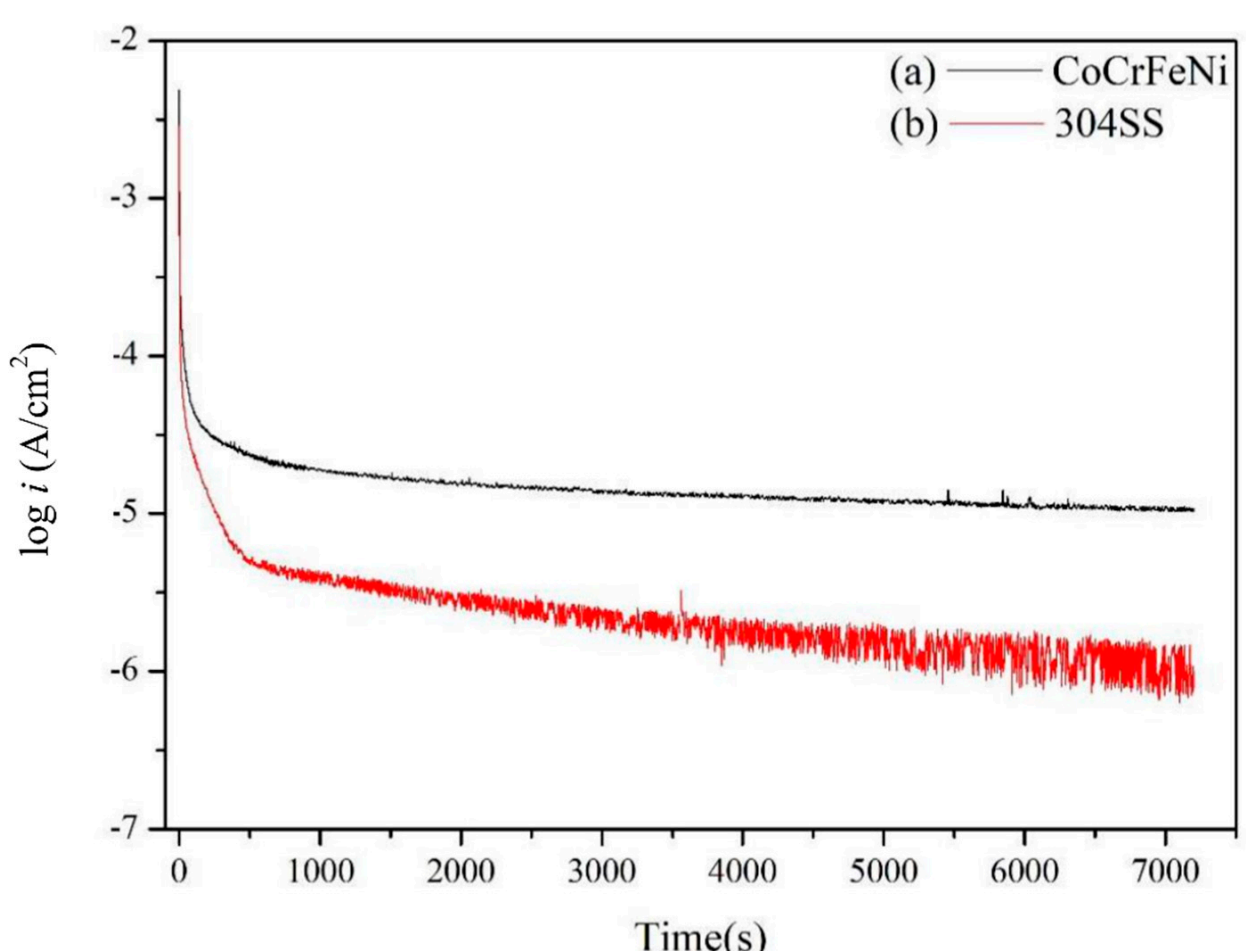
Current densities of (a) CoCrFeNi HEA and (b) 304SS at constant potential 0.6 V for 2 h.

**Figure 5:**
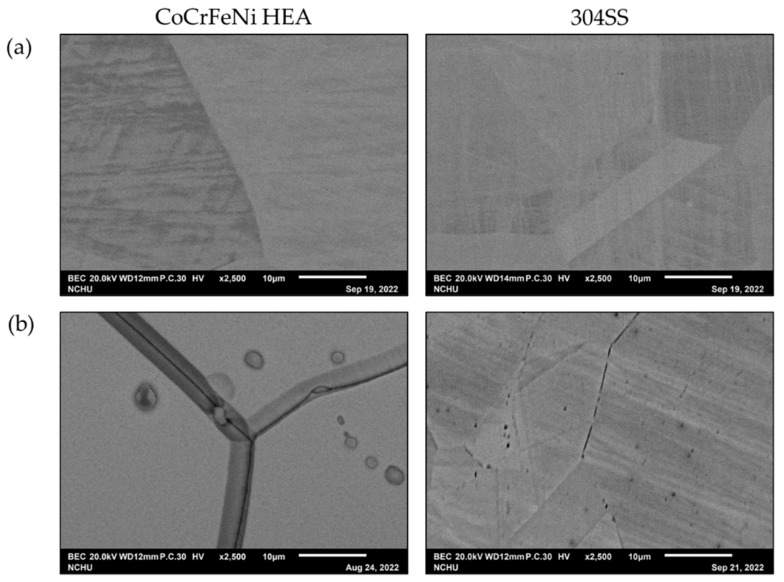
SEM observations of CoCrFeNi HEA and 304SS (**a**) before and (**b**) after potentiodynamic tests.

These two calculated reduction-oxidation (metal–metal ions) equilibrium potentials ($E_{eq}^{’}$) of CoCrFeNi and 304SS are also listed in Table 3. Similarly, $E_{eq\;304SS\;}^{’}$ is lower than $E_{eq\;CoCrFeNi}^{’}$. The potentiodynamic curves are decomposed into anodic curves and cathodic curves around the mixed potentials by Tafel region slopes and the diffusion limiting current density of O_2_ as shown in Figure 2a for CoCrFeNi HEA and Figure 2b for 304SS. Figure 2 presents the anodic curves and cathodic curves intersecting three times, thus revealing three corrosion potentials (*E_corr_*), two critical current densities (*i_crit_*), and three corrosion current densities (*i_corr_*). However, not only the greater *i_crit_*_1_ but also the wider active potential range is found for 304SS, meaning it is harder to be passivated around this region. In contrast, the anodic curves and cathodic curves only intersect one time for CoCrFeNi HEA with pre-immersion for 30 min before the test, as shown in Figure 3a, indicating that the CoCrFeNi HEA was further passivated to reduce the first critical current density (*i_crit_*_1_) to be less than the cathodic reaction one. The related corrosion characteristics are listed in Table 2, revealing less *i_corr_* and *i_pass_* than the specimen without immersion. The potentiodynamic curve of 304SS with pre-immersion for 30 min is shown in Figure 3b, similar to Figure 2b. Figure 4 shows the current density variations of CoCrFeNi HEA and 304SS at a constant potential of 0.6 V for 2 h. Consistent with the results of *i_pass_*, the current density of the latter is much less than that of the former, also indicating that the passivation film of the latter is more stable than that of the former at 0.6 V, ascribed to Fe(III) and Mn(III) with the much lower k_sp_ already formed in reactions (11), (12) and (13) while this was not yet the case for Co(III) and Ni(III) in reactions (14) and (16). The SEM observations of CoCrFeNi HEA and 304SS are shown in Figure 5a before and Figure 5b after potentiodynamic tests, indicating deeper grain boundary corrosion and more corrosion products for CoCrFeNi HEA than 304SS.

### 3.2. Characteristics of the Passivation Films by X-ray Photoelectron Spectroscopy

**Figure 6:**
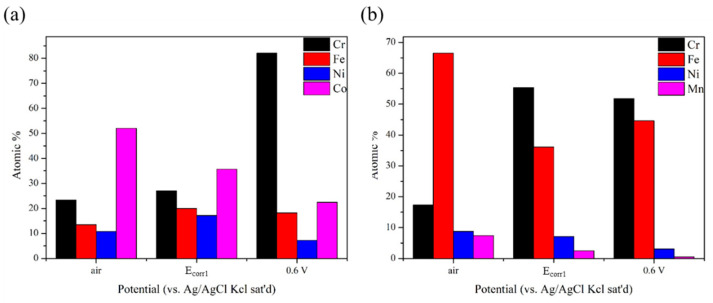
The atomic proportion variations (within 0.5% error) of metal elements in the passive films formed in air, at *E_corr_*_1_ and 0.6 V for (**a**) CoCrFeNi HEA and (**b**) 304SS specimens, derived from XPS.

Figure 6 presents the atomic proportion variations (within 0.5% error) of metal elements in the passive film for both alloys derived from XPS analyses. Quite different from the matrix of CoCrFeNi HEA, Co is the major component of passivation film in the air but decreases with the increasing potential, while Cr increases with the increasing potential and becomes the major one at 0.6 V. Ni keeps the least one. Fe in the air for HEA is a little less than that at *E_corr_*_1_ (−0.18V), which is higher than that of 304SS, especially for the immersion ones, leading to a greater ratio of stable ferric compound. Fe is the major component of passivation film in the air for 304SS, while it is replaced by Cr at *E_corr_*_1_ (−0.35V) and they are almost equal at 0.6 V. Ni and Mn are always the minor components and gradually decrease with the increasing potential, while the ratio of Mn in the passivation film is greater than that in metal matrix, resulting from *E_eq_* in reaction (10) being much lower than the others.

**Figure 7:**
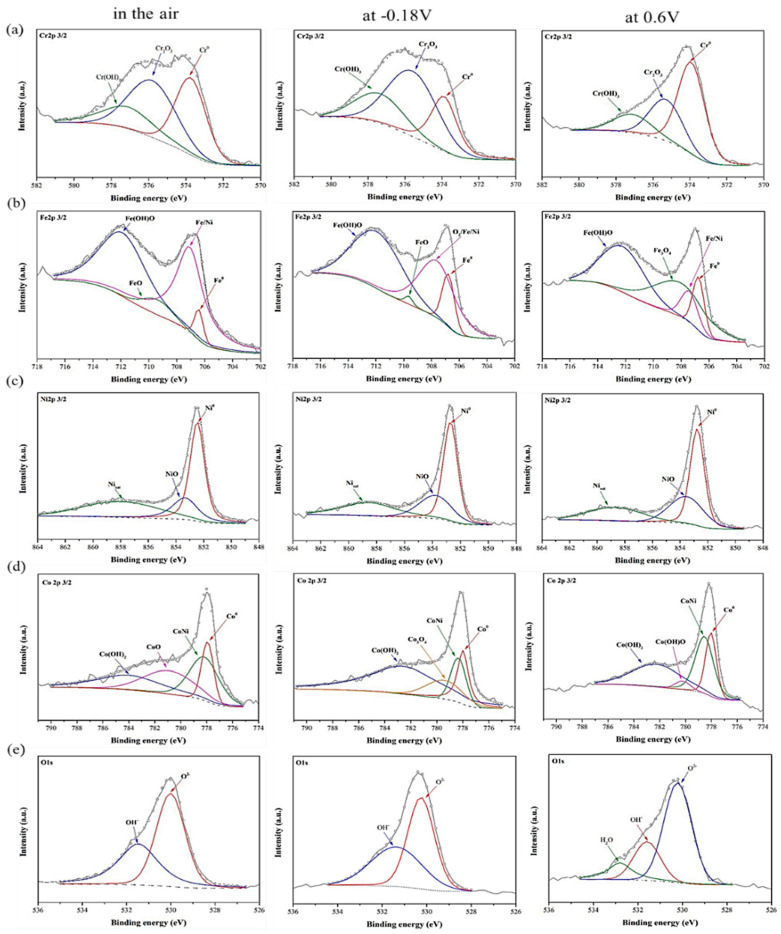
High resolution XPS spectra of the passivation films formed in the air, at E_corr1_ (−0.18V) (Ag/AgCl) and at 0.6 V(Ag/AgCl) in 0.5 M H_2_SO_4_ AAS, (**a**) Cr 2p_3/2_, (**b**) Fe 2p_3/2_, (**c**) Ni 2p_3/2_, (**d**) Co 2p_3/2_ and (**e**) O 1s on the surface of CoCrFeNi HEA.

**Figure 8:**
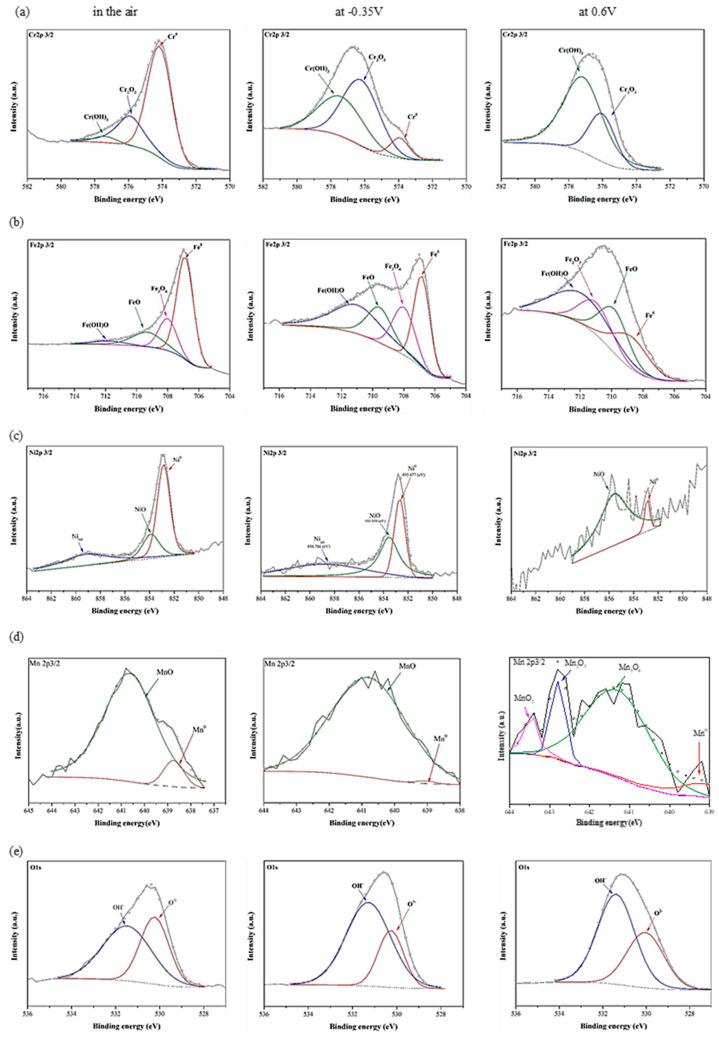
High resolution XPS spectra of the passivation films formed in the air, at E_corr1_ (−0.35V) (Ag/AgCl) and 0.6 V(Ag/AgCl) in 0.5 M H_2_SO_4_ AAS. (**a**) Cr 2p_3/2_, (**b**) Fe 2p_3/2_, (**c**) Ni 2p_3/2_, (**d**) Mn 2p_3/2_ and (**e**) O 1s on the surface of 304SS.

Figure 7 and Figure 8 show the detailed XPS results of Cr 2p_3/2_, Fe 2p_3/2_, Ni 2p_3/2_, Co 2p_3/2_, Mn 2p_3/2_, and O 1s for the CoCrFeNi HEA and 304SS passivation films in the air, at *E_corr_*_1_ and at 0.6 V passivation potential in 0.5 M H_2_SO_4_ AAS respectively. In Figure 7a and Figure 8a, the Cr 2p_3/2_ ionization is separated into several constituent peaks representing the metallic Cr^0^ (574 eV), Cr_2_O_3_ (576.1 eV), and Cr(OH)_3_ (577.2 eV). Element Cr in 304SS and CoCrFeNi HEA is primarily passivated to chromium hydroxide and oxide in 0.5 M H_2_SO_4_ AAS. Figure 7b and Figure 8b show that the Fe 2p_3/2_ ionization is split into several constituent peaks representing the metallic Fe^0^ (706.7 eV), Fe/Ni (707.2 eV), Fe_3_O_4_ (708.1 eV), FeO (709.6 eV), Fe_2_O_3_ (711.6 eV) and Fe(OH)O (711.9 eV). In Figure 7b at −0.18 V, more Fe in the CoCrFeNi HEA is oxidized to Fe(OH)O earlier than 304SS, due to the higher *E_corr_*_1_ of the former. The ferric states in both films are the main components of the iron at 0.6 V, resulting in the stable passivation regions. As shown in Figure 7c and Figure 8c, the Ni 2p_3/2_ spectra are split into three constituent peaks representing the metallic Ni^0^ (852.7 eV), NiO (853.6 eV), and Nisat (858.6 eV). The results show that the nickel on both alloys is not further oxidized to high-valence nickel such as Ni_2_O_3_ or NiO_2_ for reducing the solubility of nickel in 0.5 M H_2_SO_4_ AAS. In Figure 7d, not similar to iron, just a small part of cobalt on CoCrFeNi HEA is further oxidized into Co(OH)O at 0.6 V, a smaller k_sp_ that can also increase the corrosion resistance, revealing the enhanced passivation at potentials higher than 0.6 V. Peaks of MnO (640.8 eV) are observed on 304SS in air and at *E_corr_*_1_ (−0.35 V), then further oxidized into Mn_3_O_4_, Mn_2_O_3_ and MnO_2_, as shown in Figure 8d, basically compliant with reactions (12), (13) and (15). The O 1s spectra are separated into two or three peaks, as shown in Figure 7e and Figure 8e. The O^2-^ species (530.2 eV) are from the metal oxides. The peak at 531.5 eV represents the metal hydroxides and the peak at 532.8 eV corresponds to a little H_2_O coming from the hydrated oxides as shown in Figure 8e at 0.6 V. Cr_2_O_3_ is formed in the air for 304SS, then is hydroxylated at *E_corr_*_1_, and much more hydroxyl is formed at 0.6 V. On the other hand, the passive film for CoCrFeNi HEA is thin enough to obviously detect metal Cr, compared with 304SS where the small metal signal is detected at 0.6 V. The hydroxylation of Fe oxide occurs at 0.6 V for 304SS while it occurs at *E_corr_*_1_ for HEA, possibly by the following reactions:

Fe_3_O_4_ + FeO + 3H_2_O → 4Fe(OH)O + 2H^+^ + 2e^−^ (19)

Fe_3_O_4_ + 2H_2_O → 3Fe(OH)O + H^+^ + e^−^ (20)

A small ratio of Ni is oxidized into NiO for both CoCrFeNi HEA and 304SS. The content ratio of Cr(OH)_3_ to Cr_2_O_3_ is enhanced and the peak height (content) of Cr^0^ is reduced by the passivation for 304SS. Similar results are found for the enhanced ratio of Fe(OH)O to FeO and Fe_3_O_4_. However, they both vary scarcely for CoCrFeNi HEA. The reduced contents of Fe^0^, Cr^0^, and Ni^0^ for CoCrFeNi are much less than that for 304SS, indicating that the passivation film of the latter is thicker than that of the former at 0.6 V. The thicker the passivation film, the slower the mass transportation of the metal ion is, supporting the lower *i_pass_* of 304SS. The peaks of Ni and NiO are getting weaker by the passivation for both alloys. The content ratio of Co(OH)_2_ to CoO or Co_3_O_4_ is also enhanced at *E_corr_*_1_ as shown in Figure 7d. Although Co(OH)O was found at 0.6 V, the major phase is Co(OH)_2_, consistent with the calculated potential indicated in reactions (7) and (14). The peak ratio of OH^−^ to O^2−^ is enhanced as well at *E_corr_*_1_ and 0.6 V for 304SS, caused by the enhanced Cr(OH)_3_ and Fe(OH)O, while it is not for CoCrFeNi HEA, but a small peak of H_2_O is observed due to the hydrated oxides.

### 3.3. The Concentration of Dissolved Elements

Table 5 presents the dissolved ion concentrations of Co, Cr, Fe, Ni, and Mn for (a) CoCrFeNi and (b) 304SS specimens dynamically polarized from −1.2 V to *E_corr_*_1_ 0.25 V and 0.6 V (vs. Ref) in 0.5 M H_2_SO_4_ AAS, intermittently analyzed by ICP-MS during the polarization measurement. The *i_pass_* of CoCrFeNi shown in Figure 1 is greater than 304SS, but the total amount of ion releasing from CoCrFeNi at *E_corr_*_1_, 0.25 V or 0.6 V is significantly less than 304SS as listed in Table 5. The *E_corr_*_1_ of 304SS is −0.354 V, which is very close to the *E_eq_* of metals hydroxides for 304SS (−0.359 V). This means that reactions between metals and metal ions listed in Table 3 should be considered. In other words, the stable hydroxides of 304SS have not been completely formed yet at the *E_corr_*_1_ of 304SS since Cr and Mn hydroxides could be formed individually while the others are not yet as indicated in reactions (6), (7), (8), (9) and (10). Furthermore, the k_sp_ of Mn(OH)_2_ is much higher than those of other metal hydroxides, resulting in the large amount of Mn ion releasing from 304SS to the solution at *E_corr_*_1_ and leading to the greater increment and greater increasing ratio for Cr, Fe, Ni ions from *E_corr_*_1_ to 0.25 V before the passivation, corresponding to the greater *i_corr_*_1_ and *i_crit_*_1_, and the wider active range than CoCrFeNi shown in Figure 2a. However, the ion concentration increasing ratio from 0.25 V to 0.6 V of 304SS is much lower than that from *E_corr_*_1_ to 0.25 V since reactions (11), (12), (13) and (15) occur to form Fe(Ⅲ) and Mn(ⅢorⅣ) with much lower k_sp,_ as listed in Table 4, and further stabilize the passivation film for retarding the leaching out of Fe and Mn ions as shown in Figure 6a, corresponding to the lower *i_pass_* and *i_crit_*_2_. Obviously, most ions from 304SS were released during the polarization from −1.2 V to 0.25 V. On the other hand, the ion concentration increasing ratio of Ni or Co from 0.25 V to 0.6 V for CoCrFeNi HEA is still greater than that from *E_corr_*_1_ to 0.25 V since reactions (14) and (16) do not yet proceed and the leaching out of Co and Ni is still obvious, as revealed in Figure 6b, corresponding to the greater *i_crit_*_2_ and *i_pass_*.

### 3.4. First-Principles Calculations

**Figure 9:**
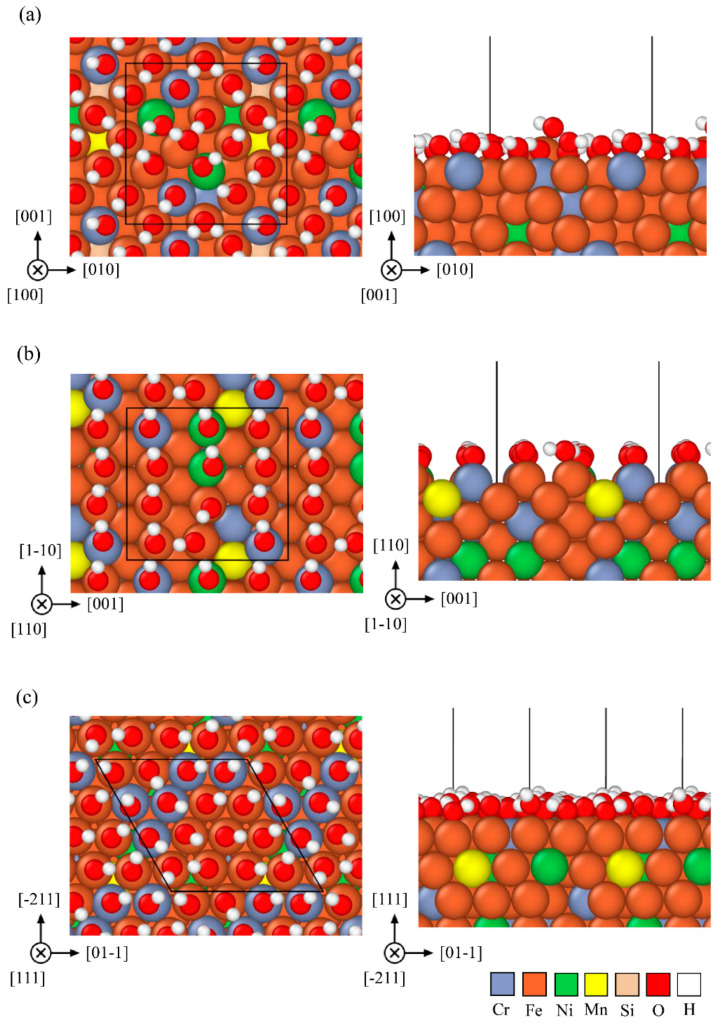
Top and side views of the atomic geometry of monolayer OH on facets (**a**) (100) (**b**) (110), and (**c**) (111) of FCC structure for 304SS specimens derived from the first-principles calculations.

**Figure 10:**
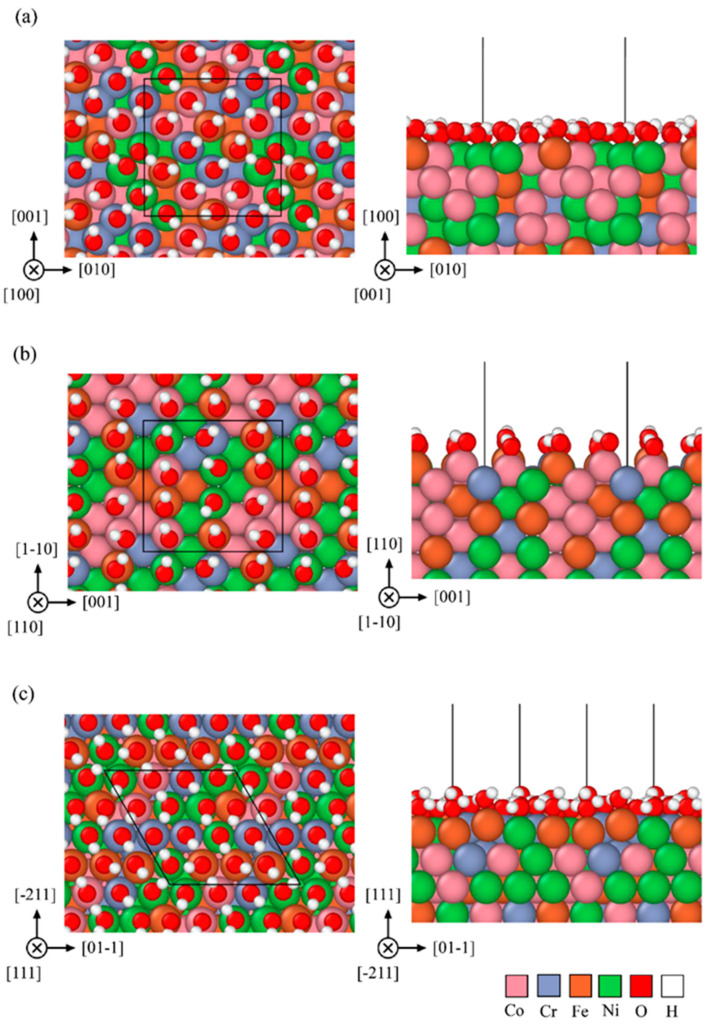
Top and side views of the atomic geometry of monolayer OH on facets (**a**) (100) (**b**) (110), and (**c**) (111) of FCC structure for CoCrFeNi specimens derived from the first-principles calculations.

Based on the composition derived from EDS data listed in Table 1, the 304SS FCC slab is composed of Cr_27_Fe_99_Ni_13_Mn_3_Si_2_ 144 atoms and the HEA one is composed of Co_36_Cr_36_Fe_36_Ni_36_ 144 atoms with 18 hydroxyl ions on the (100) facet, 12 hydroxyl ions on the (110) facet and 16 hydroxyl ions on the (111) facet. The optimized atomic geometries of (100), (110), and (111) orientations are shown in Figure 9 and Figure 10 for three facets of 304SS and HEA.

The calculated $E_{b}^{B}$ and $E_{b}^{A}$ of monolayer hydroxyl on three facets for 304SS and CoCrFeNi HEA are listed in Table 6, according to equations (4) and (5). The average *E_b_* value of 304SS is greater than that of CoCrFeNi HEA. Obviously, the *E_b_* values of monolayer hydroxyl ion adsorbed on the (100), (110), and (111) facets of CoCrFeNi HEA calculated by the first principle are less than the those of 304SS in terms of either per OH bond or per area. Meanwhile, the connecting morphology of the hydrogen bond is found to be denser and more uniform on all facets for 304SS than those for HEA, as shown in Figure 9 and Figure 10. The least *E_b_* per OH bond is 1.186 eV, found on the (111) facet of CoCrFeNi HEA and the least one per area is 0.1845 eV, found on the (110) facet of CoCrFeNi HEA. In other words, facet (111) or (110) of the CoCrFeNi HEA is less stable and preferentially corroded in AAS, consistent with the worse performances around the passivation region for equimolar CoCrFeNi HEA. It is straightforward that the higher *E_b_* of hydroxyl absorbed on the 304SS is responsible for the better corrosion resistance around the passivation region in 0.5 M H_2_SO_4_ AAS to retard the mass transportation and/or the leaching out of metal ions, leading to the lower *i_crit_*_2_ and *i_pass_* for 304SS, as listed in Table 2 or illustrated in Figure 1.

The individual k_sp_ and *E_eq_* of each metal hydroxide should be responsible for the stability of the passive film, as discussed previously by comparing with one another while neglecting the interactions of nearby elements and the geometry of hydroxyl ions adsorbed on the metal surface. In contrast, the component, the random arrangement, the coordination, and the facet orientation are all considered and calculated in general by the first-principals calculations.

## 4. Conclusions

The potentiodynamic curves are decomposed into anodic curves and cathodic curves around the mixed potentials by Tafel region slopes and the diffusion-limiting current density of O_2_. The decomposed anodic curves and cathodic curves intersect three times, thus revealing three corrosion potentials (*E_corr_*), two critical current densities (*i_crit_*), and three corrosion current densities (*i_corr_*). The potentiodynamic curve of 304SS with pre-immersion for 30 min is similar to that without pre-immersion, leading to the great amount of ion release. In contrast, the anodic and cathodic curves only intersect one time for CoCrFeNi HEA with pre-immersion for 30 min before the test, as shown in Figure 3a, indicating that the CoCrFeNi HEA was further passivated to reduce the first critical current density (*i_crit_*_1_) to be less than the cathodic reaction one, resulting in the lesser amount of ion release. Additionionally, not only the greater *i_crit_*_1_ but also the wider active potential range is found for 304SS, meaning that it is harder to be passivated around this region and reactions between metals and metal ions should be mainly considered. The Cr content and the ferric percentage should play an important role on the *i_crit_*_1_ because the k_sp_ of Cr(OH)_3_ or Fe(OH)_3_ is much less than the other metal hydroxides, including Mn(OH)_2_, Ni(OH)_2_, Co(OH)_2_, and Fe(OH)_2_. Both icrti1 and *i_corr_*_1_ of CoCrFeNi HEA are smaller than those of 304SS and are straightforwardly ascribed to the higher Cr content and greater ferric percentage of the former caused by higher *E_corr_*_1_.

The *i_pass_* of 304SS is less than that of CoCrFeNi HEA. In other words, the chemical or physical state of a passivation film on 304SS at 0.6 V should be more stable than that on CoCrFeNi, supported by the average bond energy calculation from DFT and the thicker passivation film derived from XPS, leading to the slower diffusion and/or migration rates of ions in the passivation film. According to the equilibrium potentials of reactions (11) and (16), the less Ni content in the alloy, the more stable passivation film, and the more Fe content in the alloy, the lower the potential for initiating and stabilizing passivation, also resulting in the smaller *i_pass_* and *i_crit_*_2_ for 304SS than those for CoCrFeNi HEA in 0.5 M H_2_SO_4_ AAS.

Fe is the major component of passivation film in the air for 304SS, while it is replaced by Cr at *E_corr_*_1_ and they are almost equal at 0.6 V, resulting from the much-reduced k_sp_ from the ferrous state to the ferric one. Ni and Mn are always the minor component and gradually decrease with the increasing potential due to their greater k_sp_ of Ni(Ⅱ) and Mn(Ⅱ) hydroxides. Quite different from the matrix of CoCrFeNi HEA, Co is the major component of passivation film in the air but decreases with the increasing potential since the equilibrium potential in reaction (12) is higher than 0.6 V, while Cr increases with the increasing potential and becomes the major one at 0.6 V. Ni keeps the least one. Unlike 304SS, Fe in the passive film formed at *E_corr_*_1_ is a little more than that in the air, since the *E_corr_*_1_ of CoCrFeNi HEA is higher than that of 304SS and prefers to form a more stable and higher ratio ferric compound.

Convincingly, $E_{b}^{B}$ and $E_{b}^{A}$ resulting from reactions (4) and (5) are also the key indications of thermodynamic stability for the passive film formed on alloys. The *E_b_* of monolayer hydroxyl ion adsorbed on the (111) and (110) facets of CoCrFeNi HEA calculated by the first principle is the least one in terms of per OH bond and per area, respectively, consistent with the worse performances around the passivation region such as the greater *i_crit_*_2_ and *i_pass_*. Conversely, the hydroxyl ions completely leaning on the surface and uniformly connecting with each other by hydrogen bonds leading to the greater *E_b_* can densify the passivation film to retard the mass transportation and/or the leaching out of metal ions, revealing the lower *i_crit_*_2_ and *i_pass_* for 304SS. The individual k_sp_ and *E_eq_* of each hydroxide formed on the alloy should be related to the stability during the potentiodynamic polarization while the effects of various nearby elements and the geometry of each facet coupled with a hydroxyl layer are not considered. In contrast, the component, the random arrangement of each atom, the crystal orientation, and the connecting geometry of the hydroxyl adsorbed on each facet are completely deliberated in the first principals calculation, and are likely to be more universal and precise for the evaluation around the passivation region.

## Figures and Tables

**Table 1:** Compositions of CoCrFeNi HEA and 304SS by EDS analyses (at. %).

Elements	Co	Cr	Fe	Ni	Mn	Si
304SS	-	20.65	68.65	7.50	2.12	1.08
CoCrFeNi HEA	24.36	26.85	25.18	23.61	-	

**Table 2:** Corrosion characteristics derived from polarization curves shown in Figure 1.

	CoCrFeNi HEA (without Immersion)				CoCrFeNi HEA (Immersion for 30 min)	304SS			
*E_corr_* (mV)	1st	2nd		3rd	5	1st	2nd		3rd
	−179	−116		52		−354	−87.9		183.2
*i*_corr_ (μA/cm^2^)	1st	2nd		3rd	1.4	1st	2nd		3rd
	50.2	16.2		2.14		143.5	8.4		2.3
*E_trans_* (mV)	982				963	981			
*i*_pass_ (μA/cm^2^)	7.94				4.8	1.83			
*i*_crit_ (μA/cm^2^)	1st		2nd		42.7	1st		2nd	
	152.8		35.5			390.8		3.2	

**Table 3:** The calculated reduction-oxidation equilibrium potentials of metal and metal hydroxides (*E_eq_*) by Equations (6)–(10) and that (*E’_eq_*) of metal and metal ions by Nernst equation, assuming metal ions activity at 10^−6^ M at pH = 0.47 in 0.5 M H_2_SO_4_ AAS for CoCrFeNi HEA and 304SS.

Metal-Metal hydroxides	
$E_{eq\;CoCrFeNi}^{}$ = −0.305 V (Ag/AgCl)	
$E_{eq\;304SS\;}^{}$= −0.359 V (Ag/AgCl)	
Metal-Metal ion	
Co = Co^2+^ + 2e^−^	$E_{eq}^{’}$ = −0.457V_SHE_
Cr = Cr^3+^ + 3e^−^	$E_{eq}^{’}$ = −0.864V_SHE_
Fe = Fe^2+^ + 2e^−^	$E_{eq}^{’}$ = −0.627V_SHE_
Ni = Ni^2+^ + 2e^−^	$E_{eq}^{’}$ = −0.430V_SHE_
Mn = Mn^2+^ + 2e^−^	$E_{eq}^{’}$ = −1.356V_SHE_
Si + 3H_2_O = H_2_SiO_3_ + 4H^+^ + 4e^−^	$E_{eq}^{’}$ = −1.096V_SHE_
$E_{eq\;CoCrFeNi}^{’}$ = −0.675 V_SHE_ = −0.831 V (Ag/AgCl)	
$E_{eq\;304\;SS}^{’}$ = −0.694 V_SHE_ = −0.900 V (Ag/AgCl)	

**Table 4:** Constants of solubility product k_sp_ for hydroxides of Co, Cr, Fe, Ni, and Mn [41].

Compound	k_sp_	Compound	k_sp_
Co(OH)_2_	3 × 10^−16^	Fe(OH)_2_	2 × 10^−15^
Co(OH)_3_	1 × 10^−43^	Fe(OH)_3_	6 × 10^−38^
Cr(OH)_3_	7 × 10^−31^	Ni(OH)_2_	2 × 10^−16^
Mn(OH)_2_	2 × 10^−13^	Mn(OH)_3_	1 × 10^−36^

**Table 5:** The dissolved ion concentrations (ppt) of 304SS and (CoCrFeNi HEA at the potentials of *E_corr_*_1_, 0.25 V and 0.6 V in 0.5 M H_2_SO_4_ AAS, derived from ICP-MS.

	304SS			
	Cr	Fe	Ni	Mn
*E_corr_*_1_	4.3 × 10^2^	5.6 × 10^3^	8.0 × 10^2^	5.9 × 10^3^
0.25 V	3.7 × 10^4^	1.6 × 10^5^	1.6 × 10^4^	1.2 × 10^4^
0.6 V	5.0 × 10^4^	2.1 × 10^5^	2.0 × 10^4^	1.4 × 10^4^
Increment from E_oc1_ to 0.25 V	3.6 × 10^4^	1.6 × 10^5^	1.5 × 10^4^	5.8 × 10^3^
Increase ratio from E_oc1_ to 0.25 V	85	28	19	0.99
Increment from 0.25 V to 0.6 V	1.3 × 10^3^	4.6 × 10^4^	3.7 × 10^3^	2.0 × 10^3^
Increase ratio from 0.25 V to 0.6 V	0.35	0.28	0.23	0.18
	**CoCrFeNi HEA**			
	**Cr**	**Fe**	**Ni**	**Co**
*E_corr_*_1_	3 × 10^2^	8.3 × 10^2^	3.2 × 10^2^	9.1 × 10
0.25 V	5.2 × 10^2^	3.8 × 10^3^	9.9 × 10^2^	6.9 × 10^2^
0.6 V	6.9 × 10^2^	7.3 × 10^3^	6.4 × 10^3^	6.0 × 10^3^
Increment from E_oc1_ to 0.25 V	2.2 × 10^2^	3 × 10^3^	6.8 × 10^2^	6.0 × 10^2^
Increase ratio from E_oc1_ to 0.25 V	0.72	3.57	2.14	6.57
Increment 2 from 0.25 V to 0.6 V	1.7 × 10^2^	3.5 × 10^3^	5.4 × 10^3^	5.3 × 10^3^
Increase ratio from 0.25 V to 0.6 V	0.33	0.93	5.46	7.76

**Table 6:** Average bond energy per bond ($E_{b}^{B})$ and per area ($E_{b}^{A})$ for monolayer OH on facets (100), (110), and (111) of FCC structure for 304SS and CoCrFeNi HEA.

Facets of 304SS (Cr_27_Fe_99_Ni_13_Mn_3_Si_2_)	$E_{b}^{B}$	$E_{b}^{A}$
FCC (100) (144 + 18 OH)	1.7982	0.2866
FCC (110) (144 + 12 OH)	1.9843	0.2229
FCC (111) (144 + 16 OH)	1.4342	0.2631
Average	1.7389	0.2575
**Facets of HEA (Co_36_Cr_36_Fe_36_Ni_36_)**	$E_{b}^{B}$ **(eV/OH)**	$E_{b}^{A}$ **(eV/Å^2^)**
FCC (100) (144 + 18 OH)	1.4242	0.2273
FCC (110) (144 + 12 OH)	1.6362	0.1845
FCC (111) (144 + 16 OH)	1.1862	0.2189
Average	1.4156	0.2103

## Data Availability

Not applicable.
